# Assessing Workplace Health and Safety Strategies, Trends, and Barriers through a Statewide Worksite Survey

**DOI:** 10.3390/ijerph16142475

**Published:** 2019-07-11

**Authors:** Ami Sedani, Derry Stover, Brian Coyle, Rajvi J. Wani

**Affiliations:** 1 Department of Biostatistics and Epidemiology, Hudson College of Public Health, University of Oklahoma Health Sciences Center, Oklahoma City, OK 73104, USA; 2 Division of Public Health, Nebraska Department of Health and Human Services, Lincoln, NE 68509, USA

**Keywords:** workplace health, wellness, governance, planning, barriers, survey, industry, ACA

## Abstract

Chronic diseases have added to the economic burden of the U.S. healthcare system. Most Americans spend most of their waking time at work, thereby, presenting employers with an opportunity to protect and promote health. The purpose of this study was to assess the implementation of workplace health governance and safety strategies among worksites in the State of Nebraska, over time and by industry sector using a randomized survey. Weighted percentages were compared by year, industry sector, and worksite size. Over the three study periods, 4784 responses were collected from worksite representatives. Adoption of workplace health governance and planning strategies increased over time and significantly varied across industry sector groups. Organizational safety policies varied by industry sector and were more commonly reported than workplace health governance and planning strategies. Time constraints were the most common barrier among worksites of all sizes, and stress was reported as the leading employee health issue that negatively impacts business. Results suggest that opportunities exist to integrate workplace health and safety initiatives, especially in blue-collar industry sectors and small businesses.

## 1. Introduction

Chronic diseases remain the leading cause of death and disability in the United States, as well as the leading contributor to the nation’s healthcare cost [1,2,3]. More than 150 million Americans are workers with most spending more than half of their waking time at work [4]. Maintaining a healthier workforce can lower direct costs to the business (e.g., insurance premiums and workers’ compensation claims) as well as indirect costs (e.g., absenteeism, return on investment, and worker productivity) [5,6,7,8,9]. With changes in the workforce population, chronic health conditions have become a growing concern for employees and businesses [10]. Worksite health and wellness programs offer an important population health strategy to address the increase in chronic diseases [11,12,13].

While adoption of workplace health programs have increased in the U.S. in recent years, there is still variation in uptake by business size and industries [14,15,16]. Many workplaces also lack a comprehensive, integrated approach that addresses multiple risk factors and health conditions. Successful worksite health programs are tailored to their employee population, thus making it difficult to evaluate initiatives across multiple businesses. However, all successful programs should be built on a solid foundation. According to the Centers for Disease Control and Prevention (CDC) Workplace Health Model, this foundation requires a basic organizational governance infrastructure to administer and manage health promotion activities [17].

Organizational factors are important for other aspects of worker health, including worker safety and occupational injury and illness prevention [18]. Employers have many opportunities for promoting safety and occupational injury and illness prevention at the organizational level [19]. One example is the Total Worker Health^®^ (TWH) framework, which involves organizational-level strategies aimed at integration of worksite injury prevention and health promotion activities [20]. TWH is defined as policies, programs, and practice that integrate protection from work-related safety and health hazards with promotion of injury and illness prevention efforts to advance worker well-being [21].

While many organizational approaches exist to improve worker health and safety through workplace initiatives, there is a need to better understand the adoption of these initiatives among employers. Findings have the potential to yield useful information when developing public health policies and prevention activities for improving worker health, safety, and well-being. The primary aim of this study is to assess the implementation of workplace health governance and safety strategies among worksites in the largely rural State of Nebraska, over time and by industry sector. Secondary aims include describing employer perception of barriers related to implementing workplace health strategies and employee health issues that negatively impact business. Responses on the Nebraska Worksite Wellness Surveys from 2010, 2013, and 2016 were utilized for the study.

## 2. Materials and Methods

Three point-in-time surveys were conducted among worksites in Nebraska to test our central hypothesis that the prevalence of reported health promotion and safety strategies did not vary across years or industry sectors. The sample frames for all survey years (2010, 2013, and 2016) were generated from an employer database of establishments provided by the Nebraska Department of Labor. The sample frame data included worksite name, number of employees per worksite, worksite address, and industry code. Establishments were coded and grouped using the 2-digit industry sector according to the North American Industry Classification System (NAICS) ((Health Care and Social Assistance (62); Wholesale and Retail Trade (42, 44–45); Information, Finance, and Management services (51–55); Other Services (56, 71, 72, 81); Education Services (61); Construction (23); Manufacturing (31–33); Public Administration (92); Transportation and Warehousing (48–49); All Other Sectors (11, 21, 22)).

Worksites in the sample frame were defined as establishments with a Nebraska worksite address and 10 or more employees. In order to ensure worksites of all sizes were represented in the survey data, each sample was stratified by business size: Small (10 to 49 employees), medium (50 to 199 employees), and large (200 or more employees). Disproportionate stratification was used to allow for oversampling. All large businesses in the State were included in samples (*N* = 503 in 2010; *N* = 523 in 2013; and *N* = 525 in 2016). For small and medium-sized businesses, random samples were included (*N* = 1500 in 2010 and 2013, and *N* = 2010 in 2016 for both sizes). In 2016, two priority industry sectors with low responses in the 2013 survey, ‘Construction’ and ‘Transportation and Warehousing’, were oversampled. The sampling design allowed some businesses being surveyed across all three study periods, but none of the businesses were repeated within the same time period.

The survey questions were developed by the Division of Public Health, Nebraska Department of Health and Human Services (NDHHS) in consultation with the Bureau of Sociological Research (BOSR), University of Nebraska—Lincoln. Questions were adapted from a variety of sources, and a small pilot of the survey instrument was conducted with businesses randomly selected from the sample. Because these were point-in-time surveys, worksites were asked to report current workplace practices (i.e., if they have a specific health promotion policy or program in place) and perceived barriers in each survey year. The majority of questions remained unchanged across survey years to ensure comparability over time. Survey questions pertaining to this study are in Supplementary Materials (Table S1).

The 2010, 2013, and 2016 surveys were mailed to worksites, which included a cover letter, the survey, and a postage-paid envelope. The small and medium size surveys were addressed to the business owner or manager, while the surveys for large businesses were addressed to the human resource representative. For the 2016 survey, we provided an option in 2016 for businesses to visit a website and complete the survey via a web-based questionnaire.

Results were weighted to adjust for the business size differences found between the overall sample frame and the final compilation of businesses who are represented in the completed survey data. A weighting variable was calculated by applying the appropriate sampling weights and then also adjusting for nonresponse by strata [22].

A bivariable analysis was conducted to examine the prevalence of workplace governance and safety strategies among worksites over time, by industry sector and by worksite size. Weighted percentages and confidence limits were calculated. The Rao–Scott χ^2^ statistic was used to assess differences according to year and industry sector. Effect modification was assessed by refitting the model multiple times, once for each of the main effects which was generated from the stepwise selection process. Point estimates and 95% confidence limits were calculated for multivariable analysis. The Wald χ^2^ statistic was used to compare multivariable models fit to sectors with and without a workplace governance and safety strategies. Significance levels were set at α < 0.05. All data analyses were conducted using PROC SURVEYFREQ and PROC LOGISTIC commands in SAS version 9.4 (SAS Institute Inc., Cary, NC, USA).

## 3. Results

### 3.1. Survey Responses

A total of 4784 responses were collected in 2010 (*n* = 1512; response rate: 47.4%), 2013 (*n* = 1352; response rate: 42.1%), and 2016 (*n* = 1920; response rate: 38.6%). A total of 4784 responses were collected in 2010 (*n* = 1512; response rate: 47.4%), 2013 (*n* = 1352; response rate: 42.1%), and 2016 (*n* = 1920; response rate: 38.6%). Overall, small and medium worksites were less likely to respond to the survey (response rate 39.2% and 36.3%, respectively) than large worksites (response rate: 47.3%). Over the three study periods, response rates were consistent among medium size worksites but increased among large and small worksites (*p* < 0.0001). The response rate decreased among large worksites from 54.5% in 2010 to 38.1% in 2016. Among small worksites, the response rate decreased from 43.4% in 2010 to 31.0% in 2016 (Table S2).

Table 1 presents the characteristics of participating worksites by size and industry sector grouping over the three study periods (2010, 2013, and 2016). Small (10–49 employees) and medium (50–199 employees) worksites comprised more than three-quarters of the respondents across the years. Specifically, in 2010, the majority (46%) of respondents were medium size worksites, whereas as in 2013 and 2016, the majority of respondents were small worksites (43.1% and 45.9%, respectively). Worksites in the ‘Health Care and Social Assistance’ industry sector were the most represented sector in all survey years (17–20%), followed by ‘Wholesale and Retail Trade’ (14–16%) and ‘Information, Finance, and Management Services’ (13–14%).

### 3.2. Workplace Health Governance and Planning Strategies

Increases in the weighted percentage of worksites responding “yes” to implementing workplace health governance and planning strategies were observed (Table 2). There were statistically significant differences across years in responses among questions pertaining to having an assigned coordinator for employee health promotion or wellness (*p* = 0.04), having a staff responsible for employee health promotion or wellness (*p* = 0.02), and including funding for health promotion or wellness in the worksite’s budget (in the past month) (*p* = 0.04). Among all worksites, all of the six strategies assessed were reported in less than twenty percent of worksites. All of the reported governance and planning strategies assessed significantly varied across industry sectors (*p* < 0.001). The ‘Construction’ sector reported the lowest adoption of five of the six governance and planning strategies, including having a health promotion or wellness committee (4.4%) and having a coordinator responsible for employee health promotion or wellness (6.2%). Other sectors that reported lower uptake of strategies include ‘Other Services’, ‘Transportation and Warehousing’, ‘Wholesale and Retail Trade’, and ‘Health Care and Social Assistance’. Conversely, the ‘Educational Services’ sector had the highest adoption of strategies, followed by ‘Public Administration’ and ‘Information, Finance, and Management Services’.

### 3.3. Workplace Health Governance and Planning Strategies: Multivariable Analysis

The adjusted model further illustrated the significant differences in implementation of workplace health strategies between sectors. In 2016, ‘Educational Services’ were estimated to have the greatest odds of having a coordinator responsible for employee health promotion/wellness (OR = 17.44, 95% CL: 8.85–30.56), health promotion/wellness committee (OR = 23.41, 95% CL: 13.09–41.87), staff responsible for employee health promotion/wellness (OR = 14.01, 95% CL: 8.25–23.81), funding for health promotion/wellness in budget (OR = 3.36, 95% CL: 1.97–5.71), written objectives for employee wellness/health (OR = 3.33, 95% CL: 1.95–5.69), and stated mission or goal regarding improvement or employee health status (OR = 3.32, 95% CL: 1.95–5.68), compared to ‘Wholesale and Retail Trade’ (Table 3).

### 3.4. Workplace Safety Policies

Table 4 represents the weighted proportion of worksites by industry sector that reported implementing workplace safety policies for survey years 2013 and 2016 (questions not asked in 2010). Among all industries, the most commonly reported safety policies were having a worksite safety committee (62.5%), requiring seatbelts while driving (61.5%), and having a return to work program (55.5%). Significant variation in reported safety policies across industry sectors was found among all policies assessed (*p* < 0.001). Both ‘Construction’ and ‘Transportation and Warehousing’ sectors reported the highest adoption of policies related to seatbelt use and restrictions on cell phone use and texting. Sectors reporting the highest adoption of a worksite safety committee were ‘Manufacturing’, ‘Construction’, and ‘Educational Services’. Generally, lower adoption of safety policies were reported among the sectors ‘Information, Finance, and Management Services’, ‘Health Care and Social Assistance’, ‘Wholesale and Retail Trade’, and those classified as ‘Other Services’. 

### 3.5. Workplace Safety Policies: Multivariable Analysis

The adjusted model showed that sites in the ‘Information, Finance, and Management’ sector were significantly least likely to have policies that require seatbelts while driving, promote off the job safety for employee and family, and have a return to work program for both years that these questions were asked (2013, 2016). Sites in ‘Construction’ were most likely to require seatbelts while driving in 2013 and 2016, and in 2013 they were most likely to require refraining from texting while driving. In 2013, sites in ‘Manufacturing’ were 7.88 times more likely to have a worksite safety committee than sites in ‘Wholesale and Retail Trade’ (95% CL: 2.94–21.11). In 2016, worksites in ‘Manufacturing’ were only 3.93 times more likely to have a worksite safety committee than sites in ‘Wholesale and Retail Trade’ (95% CL: 1.93–8.00) (Table 5).

### 3.6. Negative Impacts of Employee Health Issues

**Figure 1:**
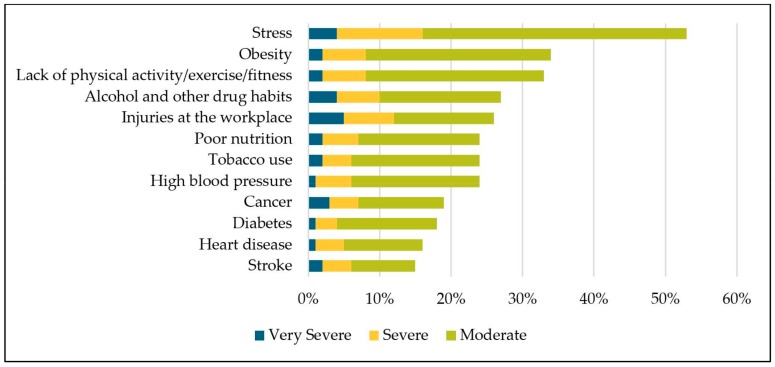
Percentage of Nebraska worksites that reported selected employee health issues negatively impacted business, 2016.

Figure 1 represents the weighted percentage of worksites that indicated specific employee health issues having a negative impact on business in 2016. Among health issues which employers noted as “very severely”, “severely”, or “moderately” having a negative impact on the worksite, stress (53%) was listed as the top issue. Obesity (34%) and lack of physical activity/exercise/fitness (33%) were the second and third most frequently cited health issues that negatively impacted business. When results were restricted to health issues that “very severely” impacted the worksite, injuries at the worksite (5%) was the most frequent health issue reported, followed by stress (4%) and alcohol/other drug habits (4%).

### 3.7. Barriers in Implementing Workplace Health and Wellness Strategies

**Figure 2:**
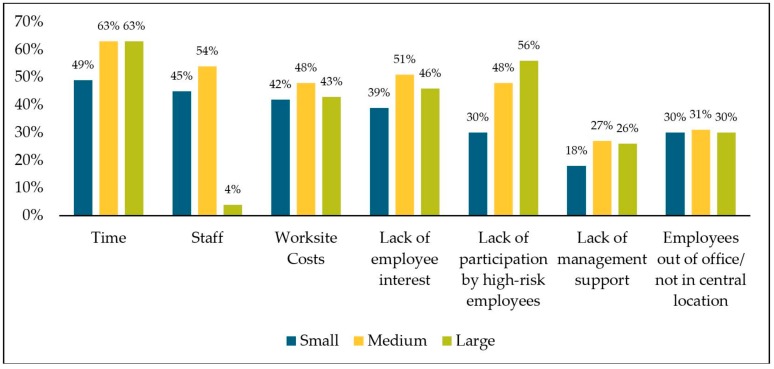
Weighted percentage of Nebraska worksites that indicated the following as barriers to implementing worksite health and wellness programs by worksite size, 2016.

Worksites indicated perceived barriers to implementing workplace health and wellness programs and policies (Figure 2). Differences in barriers were examined based on worksite size. Time constraints were the most reported barrier, regardless of worksite size. In 2016, more than half of all worksites reported time constraints as a barrier, which was lowest in small worksites (49%). Large worksites were least likely to identify staff to organize worksite health and wellness as a barrier (4%), while small worksites were least likely to identify lack of management support as a barrier (18%). More than half of large worksites reported lack of participation by high-risk employees as a barrier (56%), which was comparably lower than for small businesses (30%).

## 4. Discussion

Due to the growing burden of chronic diseases on employee health and well-being, coupled with the cost of health care coverage, businesses are adopting a wide variety of workplace health promotion initiatives. A comprehensive workplace health program consists of essential components such as: Health education, supportive physical and social environments, integration of the worksite program into the organization’s structure, linkage to related programs, and worksite screening programs [17,23]. At the same time, occupational health regulatory requirements compel employers to adopt employee safety policies aimed at injury and illness prevention. Studies highlight the important role of organizational capacity and workplace policies in the prevention of injury, illness, and chronic disease [18,24,25,26]. This study sought to learn more about the implementation of workplace health governance and planning strategies and organizational safety policies among employers in a largely rural state through a worksite survey.

When compared across survey years, we found an increase in the implementation of all the six workplace health planning and governance strategies measured. The comprehensive U.S. health care reform law was enacted in March 2010, which happened to be during the first year of our study. The Prevention and Public Health Fund (PPHF), under the ACA, includes a provision for creating employer-based wellness programs [11,27]. Peer-reviewed research on the effectiveness of the ACA’s employer-based wellness programs is limited [28]. While we did not directly assess impact of the ACA’s wellness incentives, the results of our study suggest an increase over time in the implementation of workplace health governance and planning strategies.

When results were combined over multiple study periods, we found adoption of workplace health governance and planning strategies among all worksites was relatively low (less than 20%) and varied widely across industry sectors. Higher adoption found in the ‘Educational Services’ sector was consistent with Hannon et al. who assessed workplace health capacity among mid-sized employers [29]. Comparably low implementation of governance and planning strategies was found among ‘Other Services’, ‘Construction’, and ‘Transportation and Warehousing’ industries. Studies have shown participation and availability of workplace health initiatives are generally lower among workers in blue-collar and low-wage industries [18,30,31].

Overall, the presence of selected organizational safety policies was higher than governance and planning strategies, a result consistent with similar studies [18,32]. The observed higher adoption of policies related to seatbelt use and cell phone/texting while driving in the ‘Construction’ and ‘Transportation and Warehousing’ sectors was expected considering these workers are more likely to engage in work-related travel. Among all worksites, 62.5% reported having a worksite safety committee, a similar result found in a survey among small businesses by McLellan et al. [18]. The presence of a safety committee and a return to work program was lower than expected in some sectors. For example, less than two-thirds of worksites in the ‘Health Care and Social Assistance’ sector reported having safety committees and return to work programs, despite the fact that these workers experience significant risk for occupational injuries [33].

The discordance between the adoption of governance, planning, and safety strategies and policies highlights the opportunity for integrating prevention programs at the organizational level and within specific sectors. Workers, especially in labor-intensive and blue-collar industries, face unique behavioral and occupational hazards and outcomes as evidenced by data from health behavior surveys and occupational injury surveillance [34,35,36,37,38,39]. For example, truck driving workers face environmental factors that both influence unhealthy eating patterns and excess weight gain and result in higher risks of occupational injuries and illnesses [40,41,42]. The combined health hazards and risks make workers in blue-collar worksites prime candidates for comprehensive programs which integrate injury prevention, employee safety, and worker well-being initiatives.

One approach for integrating health protection with health promotion is the TWH framework. Research supports the potential of integrated workplace approaches to improve worker health, safety, and well-being by addressing overlapping risk factors [43,44,45]. While evaluating the impacts of TWH framework is an emerging field, several studies have shown that TWH interventions can effectively address injuries and chronic diseases in specific worker populations [46,47,48,49,50]. While the current study did not evaluate specific integrated TWH interventions or programs, in our 2016 survey we found that only 15.6% of worksites reported a coordinated program for occupational health and safety with health promotion (data not shown).

Our findings on the impact of employer’s perceived health issues demonstrate a business case for TWH approaches. We found stress, obesity, physical activity, alcohol/drug use, and workplace injuries were the top five employee health issues reported by worksites which negatively affect business. These results highlight the complex and interconnected worker health dynamic which could be addressed with an integrated approach. Worksite stress, for example, is associated with negative health outcomes such as increased risk of cardiovascular disease and metabolic syndrome [51,52]. Evidence also supports the relationship between workplace injuries and chronic disease [36,53,54,55].

Our results regarding barriers suggest challenges in implementing workplace health initiatives can be attributed to both the employers and the employees, similar to other studies [14,25]. More than half of businesses stated that time constraints were a barrier to successful workplace health and wellness at their worksite. For these worksites, having a coordinator who is responsible for employee health promotion or a health promotion/wellness committee could help to provide a platform for employee engagement and collaboration to drive effective worksite health planning and implementation efforts.

Generally, small worksites were less likely to report barriers; no barrier was reported higher than 50% among small worksites. Worksite costs and time barriers were less likely to be reported among small worksites, which was a similar result in a survey among Australian workplaces [56]. There are many opportunities for workplace health and wellness programs in small businesses to be successful and well-accepted among employees. For example, the process of implementing new initiatives is comparatively less bureaucratic and easier to implement, a greater proportion of employees’ preferences may be incorporated, and employees may have greater personal accountability [14].

There were several limitations to this study. Given the self-report nature of the worksite survey, this study was susceptible to selection bias. Large worksites were more likely to complete the survey compared to small and medium sized worksites, and these large worksites may be more likely to have certain workplace health or safety initiatives. Furthermore, nonresponse rates increased over time among large and small businesses which was unexplained. This nonresponse increase could account for the significant increase in trends observed in Table 2. To mitigate selection bias, reminders were sent to potential respondents during all three years of survey collection. Weighting was also performed to adjust for the effect of nonresponses across worksite size (Table S3).

The relationship between worksite size and industry sector should be considered when interpreting the results. Generally, certain industries like ‘Construction’ tend to be smaller establishments while industries such as ‘Manufacturing’ tend to be larger. This association held true between worksite size and industry sector in our sample (*p* < 0.0001) (Table S4). Additionally, surveys were addressed to either the business owner, manager, or human resource representative, but the worksite information collected may result in misclassification if the representative was not the most appropriate respondent. Lastly, the data represents the views of a single worksite, thus caution is warranted when interpreting our results since evidence suggests that employees’ perceptions may vary from employers’ [57].

Despite these limitations, the findings can be used to guide recommendations for future workplace health and safety promotion research and practice. To our knowledge, this is the first study to describe the adoption and trends of specific workplace health governance and planning strategies using multiple point-in-time surveys. The data also fill a critical gap which no recent, publicly available, and existing data on workplace health governance and planning strategies and organizational safety policies by detailed industry sector. Lastly, our study had a relatively large sample size, especially for just one state.

The scope of this study did not allow for assessing the employee utilization of workplace health programs, thus observational studies are needed to verify the validity of these survey results. Furthermore, employee outcome data as well as employees’ perspectives need to be taken into consideration. While disparities in uptake of workplace health initiatives have been observed in this study as well as others, further research is needed to examine how to better engage high risk and underserved worker populations [14,30]. The majority of small businesses in Nebraska are in rural settings; therefore, a follow-up study on the urban and rural differences in the adoption of workplace health and safety strategies is warranted.

## 5. Conclusions

Results of this multi-year worksite survey show progress in workplace health initiatives among businesses in Nebraska. Our findings support the need for targeted approaches to building organizational capacity for comprehensive, integrated workplace health and safety programs in industries most impacted by chronic diseases and workplace injuries. The opportunity to advance worker health, safety, and well-being using TWH strategies is greater in blue-collar industries where adoption of governance and planning strategies were low. Public health practitioners should focus on how businesses can address the most common barriers to implementation relative to business size. Targeting promotion of workplace health programs in small business may be fruitful as they may face fewer obstacles.

## Figures and Tables

**Table 1:** Participating worksites by size and industry sector by year (2010, 2013, and 2016).

	2010 (*n* = 1512)		2013 (*n* = 1352)		2016 (*n* = 1920)	
	*n*	(%)	*n*	(%)	*n*	(%)
Worksite Size *						
Small (10 to 49 employees)	574	(38.0)	582	(43.1)	881	(45.9)
Medium (50 to 199 employees)	695	(46.0)	510	(37.7)	839	(43.7)
Large (more than 200 employees)	243	(16.1)	260	(19.2)	200	(10.4)
Industry Sector *						
Health Care and Social Assistance	262	(17.3)	260	(19.6)	342	(17.8)
Wholesale and Retail Trade	237	(15.7)	197	(14.8)	269	(14.0)
Information, Finance, and Management Services	189	(12.5)	188	(14.2)	236	(12.3)
Other Services	225	(14.9)	185	(13.9)	219	(11.4)
Educational Services	148	(9.8)	149	(11.2)	183	(9.5)
Construction	92	(6.1)	58	(4.4)	180	(9.4)
Manufacturing	169	(11.2)	141	(10.6)	171	(8.9)
Public Administration	113	(7.5)	80	(6.0)	130	(6.8)
Transportation and Warehousing	42	(2.8)	37	(2.8)	143	(7.5)
All Other Sectors	35	(2.3)	34	(2.6)	47	(2.5)

Note: Unweighted sample. Industry classified and grouped by NAICS 2-digit sector codes: Health Care and Social Assistance (62); Wholesale and Retail Trade (42, 44–45); Information, Finance, and Management Services (51–55); Other Services (56, 71, 72, 81); Education Services (61); Construction (23); Manufacturing (31–33); Public Administration (92); Transportation and Warehousing (48–49); All Other Sectors (11, 21, 22); * statistically significant using chi-square test, *p* < 0.0001.

**Table 2:** Weighted percentage of worksite responses to implementing workplace health strategies by industry sectors, survey years and worksite sizes.

	Health Promotion Committee *,‡	Coordinator Responsible for Employee Health Promotion *,†,‡	Staff Responsible for Employee Health Promotion *,†,‡	Funding for Health Promotion in Budget *,†	Written Objectives for Employee Health *,‡	Stated Mission or Goal Regarding Employee Health *,‡
	% (CI)	% (CI)	% (CI)	% (CI)	% (CI)	% (CI)
**Industry Sector**						
Wholesale and Retail Trade	12.6 (9.8–15.4)	15.2 (12.1–18.3)	10.9 (8.3–13.5)	10.5 (7.9–13.1)	10.6 (7.9–13.2)	10.3 (7.7–12.9)
All Other Sectors	29.9 (19.8–39.9)	27.4 (17.8–37.1)	22.6 (13.6–31.7)	27.4 (17.8–37.0)	15.1 (8.2–21.9)	11.0 (5.1–16.9)
Construction	6.2 (3.3–9.0)	4.4 (2.3–6.6)	2.6 (1.0–4.2)	5.2 (2.4–8.1)	5.0 (2.3–7.7)	2.9 (0.6–5.1)
Educational Services	64.3 (58.1–70.4)	57.8 (51.5–64.0)	47.5 (41.3–53.8)	19.6 (14.7–24.5)	22.8 (17.8–27.9)	24.0 (18.7–29.3)
Health Care and Social Assistance	16.9 (13.9–20.0)	16.8 (13.7–19.9)	16.3 (13.2–19.3)	12.9 (10.2–15.6)	11.1 (8.6–13.7)	11.2 (8.7–13.8)
Information, Finance, and Management Services	24 (20.0–28.0)	25.2 (21.1–29.3)	18.5 (15.0–22.1)	21.6 (17.8–25.4)	13.4 (10.4–16.4)	13.1 (10.2–16.0)
Manufacturing	21.6 (17.1–26.2)	23.8 (19.1–28.6)	20.7 (16.1–25.4)	19.8 (15.5–24.0)	15 (11.1–19.0)	12.3 (8.8–15.7)
Other Services	7.7 (5.4–10.1)	7.9 (5.5–10.2)	7.4 (5.0–9.7)	5.2 (3.3–7.2)	4.8 (3.0–6.6)	5.4 (3.4–7.4)
Public Administration	26.0 (19.8–32.3)	21.1 (15.4–26.9)	19.5 (13.9–25.2)	21.1 (15.2–27.0)	17.1 (11.6–22.7)	16.4 (11.0–21.9)
Transportation and Warehousing	12.7 (7.7–17.6)	14.9 (9.7–20.1)	11.1 (6.3–15.8)	8.2 (4.4–12.1)	9.5 (5.5–13.5)	8.5 (4.6–12.4)
**Year**						
2010	16.2 (14.0–18.3)	15.7 (13.6–17.8)	12.6 (10.7–14.5)	11.1 (9.3–12.9)	10.1 (8.3–11.8)	9.2 (7.5–10.9)
2013	20.0 (17.0–22.6)	19.7 (17.2–22.2)	15.7 (13.4–18.0)	13.3 (11.2–15.4)	11.9 (9.9–13.9)	11.3 (9.4–13.2)
2016	21.2 (18.9–23.5)	21.4 (19.2–23.7)	18.5 (16.4–20.7)	15.6 (13.6–17.7)	12.2 (10.5–14.0)	12.1 (10.3–13.9)
**Worksite Size**						
Small	13.8 (12.2–15.4)	14.3 (12.7–15.9)	11.5 (10.0–13.0)	9.8 (8.4–11.2)	8.2 (6.9–9.5)	7.9 (6.6–9.1)
Medium	33.1 (30.6–35.5)	30.8 (28.4–33.2)	25.9 (23.7–28.2)	21.2 (19.2–23.2)	18.1 (16.2–19.9)	17.3 (15.4–19.2)
Large	65.1 (60.3–69.8)	64.0 (59.0–69.0)	57.2 (52.3–62.0)	53.5 (48.7–58.3)	45.8 (41.1–50.4)	43.5 (38.9–48.1)
**Total**	**19.4 (18.1–20.7)**	**19.3 (17.9–20.6)**	**15.9 (14.7–17.1)**	**13.6 (12.4–14.7)**	**11.4 (10.4–12.5)**	**11.0 (10.4–12.5)**

Note: Weighted percentage (95% confidence limits); * Significant Differences for Sector, † Significant Differences for Year, ‡ Significant Differences for Size.

**Table 3:** Multivariable adjusted odds of implementing workplace health strategies stratified by year and industry sector.

Workplace Health Strategy	Industry Sector	2010	2013	2016
		Estimate (95% CL)	Estimate (95% CL)	Estimate (95% CL)
**Coordinator responsible for employee health promotion/wellness**	Wholesale and Retail Trade (Ref)	1.00	1.00	1.00
	All Other Sectors	3.01 (1.29–7.03)	3.33 (1.38–8.07)	1.70 (0.92–3.14)
	Construction	0.09 (0.02–0.38)	0.60 (0.23–1.56)	0.27 (0.12–0.61)
	Educational Services	2.87 (1.69–4.88)	11.16 (6.48–19.25)	17.44 (9.95–30.56)
	Health Care and Social Assistance	1.07 (0.65–1.76)	1.08 (0.62–1.88)	1.12 (0.75–1.66)
	Information, Finance, and Management Services	1.73 (1.08–2.79)	2.07 (1.23–3.51)	1.68 (1.14–2.47)
	Manufacturing	1.40 (0.81–2.44)	2.02 (1.07–3.81)	1.80 (1.10–2.95)
	Other Services	0.22 (0.11–0.45)	0.95 (0.54–1.68)	0.50 (0.30–0.82)
	Public Administration	1.44 (0.80–2.59)	2.23 (1.10–4.52)	1.26 (0.73–2.18)
	Transportation and Warehousing	0.22 (0.04–1.28)	1.31 (0.50–3.44)	1.43 (0.73–2.82)
**Health promotion/wellness committee**	Wholesale and Retail Trade (Ref)	1.00	1.00	1.00
	All Other Sectors	3.72 (1.58–8.77)	5.38 (2.27–12.76)	2.30 (1.25–4.26)
	Construction	0.36 (0.14–0.92)	0.723 (0.29–1.82)	0.42 (0.20–0.87)
	Educational Services	8.00 (4.65–13.75)	13.68 (7.78–24.03)	23.41 (13.09–41.87)
	Health Care and Social Assistance	1.51 (0.89–2.58)	1.21 (0.68–2.14)	1.40 (0.93–2.10)
	Information, Finance, and Management Services	2.00 (1.19–3.38)	2.17 (1.26–3.73)	2.17 (1.46–3.25)
	Manufacturing	1.55 (0.84–2.85)	2.16 (1.13–4.15)	2.02 (1.21–3.37)
	Other Services	0.37 (0.19–0.75)	0.94 (0.52–1.71)	0.60 (0.36–1.00)
	Public Administration	2.57 (1.43–4.61)	3.77 (1.92–7.38)	1.89 (1.10–3.25)
	Transportation and Warehousing	0.44 (0.10–2.01)	1.48 (0.56–3.92)	1.16 (0.54–2.48)
**Staff responsible for employee health promotion/wellness**	Wholesale and Retail Trade (Ref)	1.00	1.00	1.00
	All Other Sectors	4.14 (1.73–9.87)	2.82 (1.04–7.65)	1.93 (0.991–3.757)
	Construction	0.08 (0.01–0.55)	0.42 (0.12–1.50)	0.24 (0.09–0.632)
	Educational Services	3.52 (1.96–6.33)	9.04 (5.06–16.16)	14.01 (8.25–23.81)
	Health Care and Social Assistance	1.78 (1.03–3.06)	1.50 (0.82–2.73)	1.44 (0.94–2.20)
	Information, Finance, and Management Services	1.87 (1.07–3.25)	1.52 (0.82–2.83)	1.86 (1.22–2.85)
	Manufacturing	1.62 (0.86–3.04)	2.88 (1.47–5.64)	2.09 (1.23–3.54)
	Other Services	0.22 (0.09–0.54)	1.13 (0.60–2.13)	0.78 (0.48–1.30)
	Public Administration	2.16 (1.14–4.09)	2.79 (1.32–5.89)	1.61 (0.90–2.87)
	Transportation and Warehousing	0.15 (0.01–2.07)	1.70 (0.62–4.71)	1.25 (0.57–2.73)
**Funding for health promotion/wellness in budget**	Wholesale and Retail Trade (Ref)	1.00	1.00	1.00
	All Other Sectors	6.03 (2.51–14.52)	6.56 (2.66–16.16)	2.18 (1.12–4.25)
	Construction	0.36 (0.13–1.02)	1.20 (0.51–2.86)	0.26 (0.10–0.67)
	Educational Services	0.94 (0.41–2.12)	2.36 (1.24–4.49)	3.36 (1.97–5.71)
	Health Care and Social Assistance	1.56 (0.87–2.79)	0.91 (0.48–1.73)	1.19 (0.76–1.85)
	Information, Finance, and Management Services	2.76 (1.59–4.80)	1.92 (1.07–3.47)	2.31 (1.51–3.54)
	Manufacturing	1.86 (0.98–3.53)	3.13 (1.63–6.02)	1.55 (0.88–2.74)
	Other Services	0.27 (0.12–0.64)	0.69 (0.35–1.37)	0.50 (0.28–0.88)
	Public Administration	1.93 (0.98–3.80)	3.29 (1.60–6.77)	2.30 (1.32–4.02)
	Transportation and Warehousing	0.05 (<0.001–5.29)	0.94 (0.29–3.12)	1.05 (0.46–2.41)
**Written objectives for employee wellness/health**	Wholesale and Retail Trade (Ref)	1.00	1.00	1.00
	All Other Sectors	3.21 (1.28–8.04)	5.32 (2.11–13.43)	0.39 (0.12–1.25)
	Construction	0.19 (0.05–0.72)	1.43 (0.62–3.34)	0.28 (0.11–0.70)
	Educational Services	1.83 (0.93–3.61)	3.06 (1.64–5.73)	3.33 (1.95–5.69)
	Health Care and Social Assistance	1.31 (0.73–2.37)	0.73 (0.36–1.46)	1.05 (0.67–1.65)
	Information, Finance, and Management Services	1.47 (0.82–2.63)	1.27 (0.67–2.42)	1.17 (0.73–1.86)
	Manufacturing	1.23 (0.62–2.433)	1.94 (0.94–3.99)	1.36 (0.76–2.44)
	Other Services	0.24 (0.10–0.58)	0.93 (0.482–1.80)	0.34 (0.18–0.65)
	Public Administration	2.17 (1.12–4.19)	2.13 (0.97–4.69)	1.52 (0.83–2.77)
	Transportation and Warehousing	0.59 (0.14–2.53)	0.94 (0.27–3.23)	1.07 (0.47–2.45)
**Stated mission or goal regarding improvement or employee health status**	Wholesale and Retail Trade (Ref)	1.00	1.00	1.00
	All Other Sectors	1.56 (0.50–4.84)	3.52 (1.33–9.35)	0.47 (0.16–1.38)
	Construction	0.17 (0.04–0.70)	0.97 (0.37–2.50)	0.08 (0.01–0.40)
	Educational Services	2.35 (1.23–4.46)	2.95 (1.58–5.51)	3.32 (1.95–5.68)
	Health Care and Social Assistance	1.37 (0.76–2.48)	0.96 (0.50–1.83)	0.98 (0.61–1.56)
	Information, Finance, and Management Services	1.19 (0.64–2.20)	1.24 (0.65–2.37)	1.34 (0.84–2.13)
	Manufacturing	0.93 (0.44–1.95)	1.54 (0.72–3.30)	1.21 (0.66–2.23)
	Other Services	0.25 (0.10–0.59)	0.77 (0.39–1.53)	0.59 (0.34–1.03)
	Public Administration	1.73 (0.87–3.43)	2.41 (1.12–5.19)	1.47 (0.80–2.71)
	Transportation and Warehousing	0.32 (0.05–2.15)	0.88 (0.25–3.08)	1.13 (0.49–2.61)

**Table 4:** Weighted percentage of worksites that responded “Yes” to implementing workplace safety policies, by industry sectors, survey years, and worksite sizes.

	Require Seatbelts While Driving *,‡	Require Refrain from Talking on Cell Phone While Driving *,†,‡	Require Refrain from Texting While Driving *,‡	Promotes Off-The-Job Safety for Employee and Family *,‡	Return to Work Program *,‡	Worksite Safety Committee *,‡
	**%** (CI)	**%** (CI)	**%** (CI)	**%** (CI)	**%** (CI)	**%** (CI)
**Industry Sector**						
Wholesale and Retail Trade	67.7 (62.4–73.0)	54.5 (48.9–60.1)	60.5 (55.0–66.0)	28.8 (23.8–33.9)	59.2 (53.6–64.8)	62.7 (57.2–68.2)
All Other Sectors	76.0 (64.0–87.9)	65.1 (51.9–78.4)	66.7 (79.8–79.8)	36.5 (23.8–49.2)	68.1 (55.2–81.0)	75.5 (63.7–87.3)
Construction	91.6 (88.0–95.2)	69.9 (63.4–76.4)	82.2 (77.0–87.3)	45.3 (38.2–52.3)	73.1 (66.7–79.5)	84.6 (79.4–89.8)
Educational Services	71.8 (64.5–79.0)	63.2 (55.8–70.6)	66.7 (59.5–73.9)	42.8 (35.2–50.4)	46.6 (39.0–54.3)	83.1 (77.2–89.0)
Health Care and Social Assistance	51.6(46.2–57.1)	46.2 (40.8–51.6)	47.6 (42.2–52.9)	33.2 (28.2–38.2)	53.6 (48.2–59.1)	60.6 (55.2–66.1)
Information, Finance, and Management Services	45.1(39.0–51.1)	36.6 (30.8–42.4)	41.1 (35.1–47.0)	19.0 (14.5–23.4)	42.1 (36.1–48.1)	48.2 (42.1–54.3)
Manufacturing	74.0 (66.5–81.5)	61.6 (53.5–69.6)	64.1 (56.2–72.1)	41.4 (33.6–49.2)	72.6 (64.7–80.6)	90.4 (84.9–95.9)
Other Services	45.2 (39.3–51.1)	37.5 (31.8–43.2)	39.6 (33.9–45.3)	25.8 (20.7–30.9)	48.3 (42.4–54.1)	40.7 (35.0–46.4)
Public Administration	73.8 (65.2–82.3)	48.4 (39.0–57.8)	50.2 (40.7–59.6)	33.4 (24.6–42.1)	55.8 (46.4–65.3)	74.3 (65.5–83.1)
Transportation and Warehousing	91.5 (86.8–96.3)	88.0 (82.8–93.3)	89.4 (84.4–94.4)	49.8 (40.6–59.0)	77.8 (70.8–84.7)	66.7 (57.8–75.6)
**By Year**						
2010	56.9 (53.7–60.2)	41.7 (38.5–44.9)	-	-	-	-
2013	61.3 (58.0–64.7)	50.3 (46.9–53.7)	54.4 (51.0–57.9)	30.7 (27.6–33.8)	55.7 (52.3–59.2)	63.0 (59.7–66.4)
2016	61.9 (58.9–64.9)	51.2 (48.3–54.2)	54.7 (51.7–57.7)	32.5 (29.8–35.2)	55.2 (52.2–58.2)	62.1 (59.1–65.1)
**Worksite Size**						
Small	56.9 (54.6–59.2)	45.0 (42.7–47.2)	51.8 (49.1–54.5)	28.5 (26.1–30.9)	51.1 (48.4–53.9)	57.2 (54.5–59.9)
Medium	69.8 (67.2–72.4)	57.1 (54.4–59.9)	66.2 (63.7–68.8)	42.5 (39.8–45.3)	72.7 (70.2–75.1)	84.6 (82.6–86.5)
Large	78.0 (74.6–81.4)	62.4 (57.9–66.9)	68.3 (63.9–72.8)	61.2 (56.5–65.9)	80.4 (76.6–84.3)	89.5 (86.5–92.5)
**Total**	**61.5 (59.3–63.8)**	**50.8 48.6–53.1)**	**54.6 (52.4–56.9)**	**31.8 (29.7–33.8)**	**55.5 (53.2–57.8)**	**62.5 (60.2–64.7)**

Note: Weighted percentage (95% Confidence limits); * Significant Differences for Sector, † Significant Differences for Year, ‡ Significant Differences for Size.

**Table 5:** Multivariable regression model of implementing workplace safety policies by sector, stratified by year and industry sector.

Workplace Safety Policy	Industry Sector	2013	2016
		Estimate (95% CL)	Estimate (95% CL)
**Require seatbelts while driving**	Wholesale and Retail Trade (Ref)	1.00	1.00
	All Other Sectors	1.58 (0.51–4.96)	1.84 (0.781–4.34)
	Construction	29.49 (2.18–398.55)	3.07 (1.50–6.24)
	Educational Services	1.08 (0.59–1.98)	0.88 (0.50–1.56)
	Health Care and Social Assistance	0.36 (0.23–0.58)	0.42 (0.29–0.61)
	Information, Finance, and Management Services	0.33 (0.20–0.53)	0.23 (0.16–0.34)
	Manufacturing	1.26 (0.62–2.55)	1.518 (0.8–2.88)
	Other Services	0.35 (0.22–0.56)	0.47 (0.31–0.70)
	Public Administration	0.84 (0.41–1.73)	1.23 (0.67–2.24)
	Transportation and Warehousing	4.98 (1.11–22.43)	2.71 (1.04–7.03)
**Require refrain from talking on cell phone while driving**	Wholesale and Retail Trade (Ref)	1.00	1.00
	All Other Sectors	2.20 (0.83–5.79)	1.632 (0.842–3.162)
	Construction	2.26 (1.14–4.48)	1.395 (0.888–2.191)
	Educational Services	1.67 (0.97–2.89)	1.502 (0.892–2.529)
	Health Care and Social Assistance	0.63 (0.41–0.97)	0.745 (0.531–1.046)
	Information, Finance, and Management Services	0.54 (0.35–0.84)	0.328 (0.23–0.469)
	Manufacturing	1.12 (0.64–1.97)	1.36 (0.83–2.23)
	Other Services	0.54 (0.35–0.84)	0.62 (0.43–0.88)
	Public Administration	0.65 (0.35–1.20)	0.70 (0.44–1.13)
	Transportation and Warehousing	8.34 (2.22–31.32)	4.69 (1.95–11.26)
**Require refrain from texting while driving**	Wholesale and Retail Trade (Ref)	1.00	1.00
	All Other Sectors	2.29 (0.74–7.10)	1.26 (0.65–2.45)
	Construction	8.07 (2.37–27.46)	1.91 (1.15–3.18)
	Educational Services	1.11 (0.64–1.94)	1.46 (0.85–2.52)
	Health Care and Social Assistance	0.39 (0.25–0.60)	0.65 (0.46–0.92)
	Information, Finance, and Management Services	0.44 (0.28–0.69)	0.31 (0.22–0.44)
	Manufacturing	0.67 (0.38–1.19)	1.40 (0.83–2.35)
	Other Services	0.40 (0.25–0.62)	0.55 (0.38–0.79)
	Public Administration	0.38 (0.20–0.70)	0.65 (0.40–1.06)
	Transportation and Warehousing	5.76 (1.46–22.70)	4.58 (1.78–11.77)
**Promotes off-the-job safety for employee and family**	Wholesale and Retail Trade (Ref)	1.00	1.00
	All Other Sectors	1.89 (0.85–4.24)	1.39 (0.77–2.53)
	Construction	2.20 (1.22–3.96)	1.96 (1.28–3.00)
	Educational Services	1.58 (0.97–2.56)	2.14 (1.34–3.42)
	Health Care and Social Assistance	1.08 (0.712–1.63)	1.06 (0.76–1.48)
	Information, Finance, and Management Services	0.44 (0.27–0.73)	0.54 (0.37–0.79)
	Manufacturing	1.72 (1.01–2.92)	1.62 (1.02–2.55)
	Other Services	0.83 (0.53–1.28)	1.03 (0.72–1.49)
	Public Administration	0.93 (0.50–1.74)	1.37 (0.85–2.21)
	Transportation and Warehousing	2.87 (1.39–5.92)	2.15 (1.20–3.86)
**Return to work program**	Wholesale and Retail Trade (Ref)	1.00	1.00
	All Other Sectors	2.58 (0.88–7.60)	4.09 (1.50–11.16)
	Construction	3.24 (1.55–6.78)	1.23 (0.77–1.98)
	Educational Services	0.65 (0.40–1.06)	0.61 (0.37–1.01)
	Health Care and Social Assistance	1.07 (0.70–1.61)	0.53 (0.37–0.74)
	Information, Finance, and Management Services	0.56 (0.37–0.85)	0.43 (0.30–0.63)
	Manufacturing	3.27 (1.67–6.41)	1.29 (0.76–2.19)
	Other Services	0.95 (0.63–1.43)	0.56 (0.39–0.81)
	Public Administration	1.24 (0.66–2.32)	0.64 (0.39–1.04)
	Transportation and Warehousing	4.46 (1.62–12.30)	1.68 (0.82–3.46)
**Worksite safety committee**	Wholesale and Retail Trade (Ref)	1.00	1.00
	All Other Sectors	1.31 (0.54–3.19)	2.23 (1.04–4.72)
	Construction	2.54 (1.22–5.30)	2.61 (1.48–4.59)
	Educational Services	3.21 (1.71–6.04)	2.39 (1.28–4.50)
	Health Care and Social Assistance	0.68 (0.45–1.03)	0.73 (0.52–1.03)
	Information, Finance, and Management Services	0.60 (0.39–0.92)	0.38 (0.27–0.55)
	Manufacturing	7.88 (2.9–21.11)	3.93 (1.93–8.00)
	Other Services	0.36 (0.24–0.53)	0.36 (0.25–0.51)
	Public Administration	1.86 (0.91–3.78)	1.29 (0.76–2.18)
	Transportation and Warehousing	1.10 (0.50–2.41)	1.17 (0.61–2.26)

## Supplementary Materials

The following are available online at https://www.mdpi.com/1660-4601/16/14/2475/s1, Table S1: Survey questions on workplace health governance, planning, and safety policies., Table S2. Worksite sizes related to response rates., Table S3. Testing responses of worksites associated over time with nonresponse., Table S4. Industry sector by worksite size among respondents.
