# Supplementary material for: Assessing Workplace Health and Safety Strategies, Trends, and Barriers through a Statewide Worksite Survey
Source: Int J Environ Res Public Health. 2019 Jul 11;16(14):2475. doi: 10.3390/ijerph16142475 (PMC6678341; doi:10.3390/ijerph16142475)
Supplement: Supplementary file 1 [file ijerph-16-02475-s001.pdf]

Supplementary Materials

Supplementary Table S1. Survey questions on workplace health governance, planning, and safety policies.

The following questions are a selection of questions from a larger 8 page worksite health and wellness questionnaire for employers. A copy of the entire questionnaire can be requested by contacting the contact author.

**Policies supporting health promotion**
**Our worksite:**
Has a health promotion or wellness committee.
Has a coordinator that is responsible for employee health promotion or wellness.
Has staff that are responsible for employee health promotion or wellness.
Has included funding for health promotion or wellness in the budget *in the past month*.
Has written objectives for employee wellness or health.
Has a stated mission or goal for our company regarding the improvement of the health status of employees.
Has coordinated programs for occupational health and safety with program for health promotion and wellness
**Policies regarding worker safety**
**Our worksite:**
Has policies that require employees to wear seatbelts while driving a car or operating a moving vehicle while on company business.
Has policies that require employees to wear seatbelts while riding in a car or moving vehicle while on company business.
Has policies that require employees to refrain from talking on cellular phones while driving a car or operating a moving vehicle while on company business.
Has a policy/policies that require employees to refrain from texting on cellular phones while driving a car or operating a moving vehicle while on company business.
Promotes off-the-job safety for the employee and their family on topics such as fall prevention, car seat/seatbelt use and/or poisoning prevention.
Has a return to work program for injured employees.
Has a worksite safety committee.
Injuries at the workplace

Supplementary Table S2. Worksite sizes related to response rates. [a]

Worksite size	2010	2013	2016	P-value
**Responses**	N			
Small (10 to 49 employees)	651	582	623	<0.001
Medium (50 to 99 employees)	587	510	785	
Large (more than 200 employees)	274	260	200	
Total	1512	1352	1608	
**Non-responses**				
Small (10 to 49 employees)	849	918	1387	0.003
Medium (50 to 99 employees)	913	990	1225	
Large (more than 200 employees)	229	263	325	
Total	1991	2171	2937	

[a] unweighted data

[b] data do not include responses and nonresponses for construction oversample and transportation and warehousing oversample strata from the 2016 survey.

Supplementary Table S3: Testing responses of worksites associated over time with nonresponse.[a]

Worksite size	Survey year						P-value
	2010		2013		2016 [b]		
	Responses	Non-responses	Responses	Non-responses	Responses	Non-responses	
Small	651 (43%)	849 (56.6%)	582 (38.8%)	918 (61.2%)	623 (31.0%)	1387 (69.0%)	<0.0001
Medium	587 (39%)	913 (60.9%)	510 (34.0%)	990 (66.0%)	785 (39.1%)	1225 (60.9%)	<0.0001
Large	274 (54%)	229 (45.5%)	260 (49.7%)	263 (50.3%)	200 (38.1%)	325 (61.9%)	<0.0001

[a] unweighted data

[b] data do not include responses and nonresponses for construction oversample and transportation and warehousing oversample strata from the 2016 survey.

Supplementary Table S4: Industry sector by worksite size among respondents. [a]

	Small		Medium		Large		Total
**Industry Sector**	n	%	n	%	n	%	
Health Care and Social Assistance	286	33%	460	53%	118	14%	864
Wholesale and Retail Trade	374	53%	232	33%	97	14%	703
Information, Finance, and Management Services	275	45%	231	38%	107	17%	613
Other Services	362	58%	224	36%	43	7%	629
Educational Services	91	19%	304	63%	85	18%	480
Construction	238	72%	74	22%	18	5%	330
Manufacturing	111	23%	228	47%	142	30%	481
Public Administration	91	28%	177	55%	55	17%	323
Transportation and Warehousing	137	62%	73	33%	12	5%	222
All Other Sectors	61	53%	33	28%	22	19%	116
NEC or Unknown	11	48%	8	35%	4	17%	23
**Total**	**2037**	**43%**	**2044**	**43%**	**703**	**15%**	**4784**

[a] unweighted data
